# Risk factors of disturbed sleep phases to posterior circulation cerebral infarctions: A single-center retrospective study

**DOI:** 10.1097/MD.0000000000035479

**Published:** 2023-10-13

**Authors:** Lu Liu, Weiping Wang, Nan Gao, Tongle Jia, Li Guo, Liying Geng, Yaning Ma

**Affiliations:** a Department of Neurology, Baoding No.1 Central Hospital, Baoding, Hebei, China; b Department of Neurology, The Second Hospital of Hebei Medical University, Shijiazhuang, Hebei, China; c Department of Cardiology, Baoding No.1 Central Hospital, Baoding, Hebei, China.

**Keywords:** anterior circulation cerebral infarction, awake phase, non-rapid eye movement 3 phase, posterior circulation cerebral infarction, sleep phases disturbance

## Abstract

Posterior circulation stroke differs from anterior circulation stroke in terms of etiological, clinical, and prognostic properties. Sleep architecture is impaired in patients with acute stroke, which may correlate with disease severity and outcome, and the correlation between the location of cerebral infarction (CI) and sleep phase disturbance remains unknown. This study aimed to assess the correlation between disturbed sleep phases in CI and posterior circulation cerebral infarction (PCCI). We retrospectively enrolled 192 patients with first-onset acute CI, who were assigned to the anterior circulation cerebral infarction (n = 101) and PCCI (n = 91) groups. The polysomnograms in both groups were analyzed by phase. The proportions of sleep phases were significantly different between the 2 groups (*P* < .05). The awake (W) and non-rapid eye movement 3 (N3) phases were independently associated with PCCI in multivariate analysis. The W phase may be a risk factor for PCCI (odds ratio = 1.60, 95% CI 1.30–1.97), while the N3 phase may be a protective factor for PCCI (odds ratio = 0.498, 95% CI 0.353–0.703). This study demonstrated that CI causes different degrees of sleep phase disturbances, and the percentages of W and N3 phase disturbances were independent factors associated with PCCI. The former was a risk factor, whereas the latter was a protective factor. This study demonstrated the correlation between cerebral infarction and sleep phase disturbances from a new perspective and suggested that cerebral infarcts may alter the structure of sleep.

## 1. Introduction

Cerebral infarction (CI), also known as stroke or cerebrovascular accident, refers to “any objective evidence of permanent brain, spinal cord, or retinal cell death attributed to a vascular etiology based on pathological or imaging evidence, with or without the presence of clinical symptoms”.^[1]^ Stroke is the main cause of adult disability worldwide and affects 800,000 Americans each year.^[2]^ According to the World Health Organization, stroke caused 5.7 million deaths and 16 million first-time occurrences in 2005, and these figures are projected to reach 7.8 million and 23 million by 2030, respectively.^[3]^ Posterior circulation stroke differs from strokes of the anterior circulation in etiological, clinical, and prognostic properties.^[4]^

Sleep architecture is a term used to define the basic structural organization of normal sleep and encompasses 5 phases, including the awake (wake-after-sleep-onset, W), non-rapid eye movement sleep (N, divided into phases N1-3, representing a continuum of relative depth), and rapid eye movement sleep (REM) phases.^[5]^ A sleep episode begins with a short period of N1 progressing through N2, followed by non-rapid eye movement 3 (N3), and finally to REM.^[6,7]^ Sleep architecture is impaired in acute stroke patients, which may correlate with disease severity and outcome. Stroke patients have a reduced total sleep time (TST) and prolonged wakefulness during sleep from previous researches. Specifically, acute ischemic stroke is followed by a considerable reduction in the REM phase in humans.^[8,9]^

Although the relationship between sleep disorders and stroke has been well studied, as reflected by the above reports, the correlation between the location of the cerebral infarction and sleep phases remains unknown. Therefore, the present study aimed to assess the correlation between disturbed sleep phases in patients with CI and posterior circulation cerebral infarction (PCCI) and to identify the risk factors for PCCI.

## 2. Methods

### 2.1. Patients

This single-center retrospective study consecutively enrolled patients with a history of first-onset stroke who were treated in the Neurological Department of Baoding No.1 Central Hospital between January and September 2019. Based on the exclusion criteria, 192 patients were included in this study. This study was approved by the Ethics Committee of Baoding No.1 Central Hospital (approval number [2017]003) and the requirement for informed consent was waived because of its retrospective nature.

#### 2.1.1. Inclusion criteria.

1. Stroke confirmed by cranial diffusion-weighted imaging in accordance with The Chinese Guidelines for Diagnosis and Treatment of Acute Ischemic Stroke 2018^[10]^.
2. Simple anterior circulation cerebral infarction (ACCI) or PCCI.
3. Within 1 week after the stroke event onset and had no previous stroke history.
4. National Institutes of Health Stroke Scale score ≤ 5 points,^[11]^ and modified ranking scale score ≤ 2 points^[12]^; and
5. Age from 45 to 75 years.

#### 2.1.2. Exclusion criteria.

1. No polysomnography within 1 week of CI onset.
2. History of cerebral hemorrhage, subarachnoid hemorrhage, brain trauma, in-hospital detected intracranial tumors, or cerebral arteriovenous malformations.
3. Concurrent airway-related diseases such as primary snoring and upper airway resistance syndrome, and sleep-related diseases such as narcolepsy and restless legs syndrome.
4. Infection or treatment with anti-inflammatory drugs and immunosuppressants during hospitalization.
5. Preexisting heart or lung disease, thyroid disease, immune system, or tumor.

Patient grouping was based on MRA, magnetic resonance imaging, and diffusion-weighted imaging completed within 72 hours. Individuals with anterior and posterior circulation cerebral infarctions were assigned to ACCI and PCCI groups, respectively.^[4]^

### 2.2. Evaluation parameters

All reserved subjects were monitored overnight on a polysomnography system (Alice 5, Philips Wellcome, China) in the otolaryngology department before the study only once within the first week of stroke onset because of somnipathy, according to the American Sleep Medicine Association sleep diagnosis^[13]^ and the international 10 to 20 system.^[14]^ The data were assessed by an attending physician in the department of otolaryngology. Sleep phases and related events were manually analyzed, including TST and the sleep times of the W (awake), REM, N1 + 2 (non-rapid eye movement 1 + 2), and N3 (non-rapid eye movement 3) stages. The percentage of each phase of the total sleep time was then calculated.

### 2.3. Statistical methods

Since the total sleep time of each patient was different, this study focused on the relationship between the sleep phase disturbance and the cerebral infarction location. The percentage of each sleep phase in the total sleep time of each patient was counted using measurement data. The Shapiro–Wilk test was used to check the normality of the data, and normally distributed data were expressed as mean ± standard deviation and compared using independent samples *t* test, while abnormal distribution was expressed as median (25%,75% percentage) and compared using the Mann–Whitney *U* test nonparametric test. Count data are expressed as percentages and compared using the chi-square test.

Univariate and multivariate logistic regression analyses were performed to assess the correlation between sleep phase disturbance and cerebral infarction location. We define the group as a dependent or object variable. The ACCI and PCCI groups were defined as 0 and 1, respectively. Statistical significance was set at *P* < .05. Statistical analysis was performed using SPSS 24.0 (IBM Corp., Armonk, New York). Through multivariable logistic regression analyses, the *P* value for each patient was reserved, the receiver operating characteristic curve (ROC) was drawn using STATA 13.0 (Stata Corp of the United States), and a calibration test was performed using R 4.3.0 (r-project org).

## 3. Results

### 3.1. Patient characteristics

**Figure 1.**
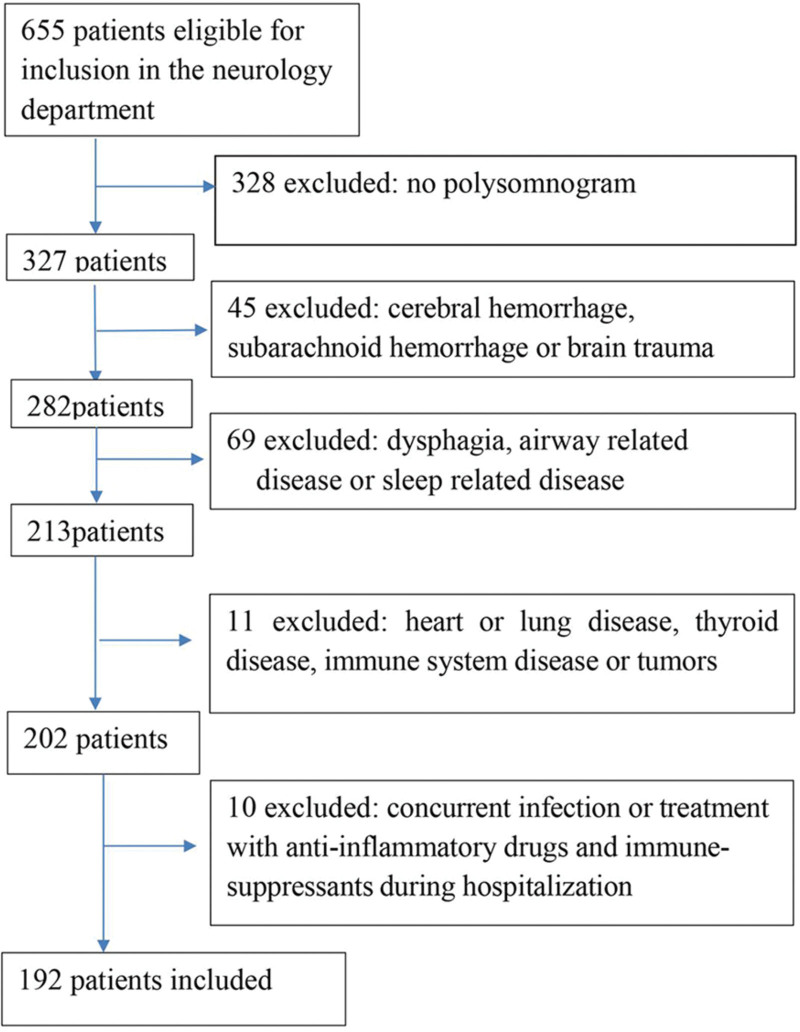
Patient flow diagram.

In total, 655 patients were enrolled in this study. Based on the eligibility criteria, 192 patients were included, with 101 in the ACCI group and 91 in the PCCI group (Fig. 1). The ACCI group included 51 males and 50 females, aged 56.25 ± 7.40 years. Among them, 71 patients had basal ganglia infarction, 2 had frontal lobe infarction, 22 had internal capsule infarction, 2 had temporal lobe infarction, and 4 had infarct lesions in multiple locations. The PCCI group comprised 47 males and 44 females. Among these patients, 60 had brainstem infarction, 12 had occipital infarction, 15 had cerebellar infarction, 3 had thalamic infarction, and 1 had infarct lesion in multiple locations. There were no statistically significant differences between the ACCI and PCCI groups in baseline patient characteristics, including age, sex, body mass index, infarct size, and general data of past medical history (all *P* > .05), as shown in Table S1 (see Table S1, Supplemental Digital Content, http://links.lww.com/MD/K209, which demonstrates general information in ACCI and PCCI patients).

### 3.2. Percentages of various phases in the ACCI and PCCI groups

Since the total sleep time of each patient was different, this study focused on the relationship between the changes in sleep phases and the cerebral infarction location. The percentage of each sleep phase in the total sleep time of each patient was counted using measurement data. The data of each phase were normally distributed and showed significant differences between the ACCI and PCCI groups (all *P* < .05). The W phase difference between the 2 groups were (13.97 ± 4.81% vs 24.31 ± 1.17%, *P* < .001). The REM phases between the 2 groups were (14.93 ± 3.91% vs 18.83 ± 4.29%, *P* < .001). The phases of N1 plus N2 (N1 + N2) between the 2 groups were (73.62 ± 6.75% vs 78.84 ± 6.56%, *P* < .001). The N3 phase percentages between the 2 groups were (11.85 ± 3.09% vs 4.70 ± 2.33%, *P* < .001). The data are summarized in Table 1.

**Table 1:** Percentages of various phases in the ACCI and PCCI groups.

Sleep phase (%)	ACCI, n = 101	PCCI, n = 91	*P* value
W	13.97 ± 4.81	24.31 ± 1.17	<.001
REM	14.93 ± 3.91	18.83 ± 4.29	<.001
N1 + 2	73.62 ± 6.75	78.84 ± 6.56	<.001
N3	11.85 ± 3.09	4.70 ± 2.33	<.001

ACCI = anterior circulation cerebral infarction, N1 = non-rapid eye movement 1, N2 = non-rapid eye movement 2, N3 = non-rapid eye movement 3, PCCI = posterior circulation cerebral infarction, REM = rapid eye movement sleep, W = wake.

### 3.3. Related factors of sleep phases to PCCI

**Figure 2.**
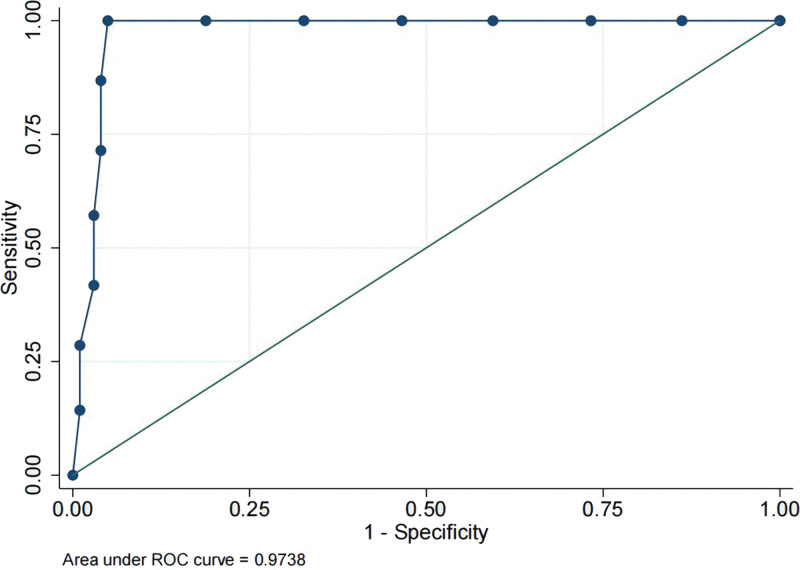
The ROC curve of the model. ROC = receiver operating characteristic.

**Figure 3.**
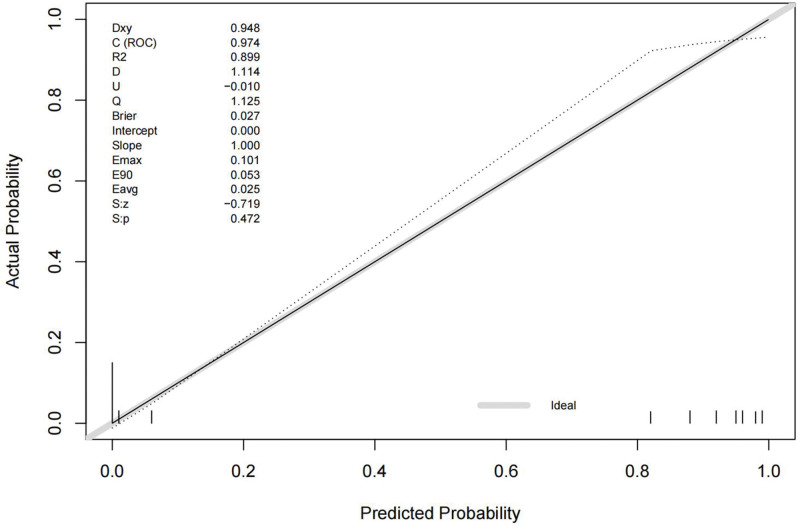
The calibration curve of the model.

In the univariate analysis, the W (odds ratio [OR] = 2.103, 95% confidence interval [95% CI] was 1.694–2.611; *P* = 0), REM (OR = 1.252, 95% CI was 1.156–1.356; *P* = 0), N1 + N2 (OR = 1.122, 95% CI = 1.017–1.176, *P* = 0), and N3 (OR = 0.39, 95% CI was 0.285–0.534; *P* = 0) phase percentages were significantly associated with PCCI. In the multivariate analysis, only the W and N3 phase percentages were significantly associated with PCCI. The W phase was a risk factor for PCCI (OR = 1.60, 95% CI was 1.30–1.97) and the N3 phase was a protective factor against PCCI (OR = 0.498, 95% CI was 0.353–0.703). The data are summarized in Tables 2 and 3. The ROC curve of the model is shown in Figure 2. The area under the ROC curve was 0.974, and the optimal cutoff value was 0.4419888. The calibration curve is shown in Figure 3. The *P* value for the calibration test was .472, the maximum offset (Emax) was 0.101, and the average offset (Evag) was 0.025.

**Table 2:** Univariable logistic regression analyses of the associations of cerebral infarction (PCCI vs ACCI) with phases in sleep architecture.

Sleep phase (%)	Univariable analysis			
	β	OR	OR 95% CI	*P* value
W	0.743	2.103	1.694–2.611	<.001
REM	0.225	1.252	1.156–1.356	<.001
N1 + 2	0.115	1.122	1.017–1.176	<.001
N3	−0.941	0.39	0.285–0.534	<.001

95% CI = 95% confidence interval, N1 = non-rapid eye movement 1, N2 = non-rapid eye movement 2, N3 = non-rapid eye movement 3, OR = odds ratio, REM = rapid eye movement sleep, W = wake.

**Table 3:** Multivariable logistic regression analyses of the associations of cerebral infarction (PCCI vs ACCI) with phases in sleep architecture.

	Multivariable analysis				
	Sleep phase (%)	β	OR	OR 95% CI	*P* value
Model 1	W	0.743	2.103	1.694–2.611	<.001
	Con.	−14.782	0	-	0
Model 2	W	0.47	1.60	1.30–1.969	<.001
	N3	−0.696	0.498	0.353–0.703	<.001
	Con.	−4.765	2.594	-	.066

95% CI = 95% confidence interval, ACCI = anterior circulation cerebral infarction, Con.: constant quantity, N1 = non-rapid eye movement 1, N2 = non-rapid eye movement 2, N3 = non-rapid eye movement 3, OR = odds ratio, PCCI = posterior circulation cerebral infarction, REM = rapid eye movement sleep, W = wake.

## 4. Discussion

A normal sleep process usually starts with a brief phase of N1, followed by phases N2 and N3, and finally enters REM sleep phase^[6]^. During the entire sleep process, the N and REM phases interchanged every 90 to 120 minutes. Normal sleep architecture includes 2% to 5% N1, 45% to 55% N2, 20% to 25% N3, and 20% to 25% REM.^[8–10]^ Multiple studies have shown that, under abnormal sleep states, the profile of sleep architecture changes significantly, especially in the N3 and REM phases.^[15–22]^ A meta-analysis revealed that control patients have better sleep quality than stroke patients: higher sleep efficiency (84% vs 75%), longer TST (340.33 vs 309.4 minutes), and less wake-after-sleep-onset (53.8 vs 97.2 minutes). Patients with stroke spend more time in the N1 phase (13% vs 10%) and less time in the N2 phase (36% vs 45%) and slow-wave sleep cycle (10% vs 12%). No group dissimilarities were detected during the REM phase.^[23]^ All-night sleep monitoring of 86 patients with acute CI was conducted, and TST, sleep efficiency, and REM phase in patients with acute CI were significantly reduced (*P* < .01) compared with the control values, while the awakening period was drastically increased (*P* < .01). As shown above, sleep phases in patients with cerebral infarction may change compared to those in normal individuals.

In our study, consistent with previous research, the sleep-wake system was affected. The REM/TST ratios in the ACCI and PCCI groups were both lower than the normal values (20%–25%).^[24–27]^ The N1 + N2/TST ratios in the ACCI and PCCI groups were both higher than the normal values,^[24–27]^ suggesting that both groups had prolonged presleep and shortened deep sleep. The N3/TST ratios in the ACCI and PCCI groups were both lower than the percentage of N3/TST ratio in normal sleep.^[24–27]^

Zhang et al^[28]^ conducted sleep evaluation and sleep breathing monitoring in 258 patients with CI and found that subcortical and brainstem infarctions were more prone to sleep architecture disturbances than those in other locations. The architecture of human sleep and wakefulness is regulated by the frontal lobe, brainstem reticulum inhibition zone, orbital cortex, ascending reticulum system, basal ganglia, thalamus, and the hypothalamus. When a stroke damages the above regions, the normal sleep-wake system is affected, causing sleep architecture disturbance.^[29,30]^ It was found that patients with cerebral cortical infarction have more serious sleep disturbances, which may be related to the distribution of sleep-related nerve architecture in the cerebral cortex.^[31]^ In addition, after CI, the synthesis/release of neuropeptides and neurotransmitters such as melatonin and serotonin is impaired.^[32]^ The sequelae of CI cause great psychological stress in patients, and certain therapeutic drugs,^[33,34]^ local muscle tension, numbness, and weakness can also affect sleep architecture. Therefore, sleep phase disturbances may have some specific correlation with cerebral infarction location.

The purpose of the current study was to establish a multi-factor logistic regression model and determine which sleep phase disturbance has a specific correlation with cerebral infarction location using it as a clinical entry point for targeted treatment.

As shown above, due to the potentially strong correlations among independent variables, the stepwise method was used to establish a multi-factor logistic regression model. The model eventually revealed that the W and N3 phases of sleep were independent factors predicting PCCI, which further illustrates that sleep architecture alterations of the W and N3 phases may be related to the circulatory distribution of cerebral infarction. The W phase was a risk factor for PCCI (OR = 1.60, 95% CI 1.30–1.97). The clinical significance is that when the percentage of the W phase increases by 1%, the apparent probability of PCCI is 1.6 times that of ACCI. N3 phase was a protective factor for PCCI (OR = 0.498, 95% CI 0.353–0.703). The clinical significance is that when the percentage of the N3 phase increases by 1%, the apparent probability of PCCI could decline by 50.2% compared with ACCI. As a result, in future studies, we could intervene in the sleep phases of patients to reduce the incidence of critical cerebral infarctions. The model showed good discrimination because the area under the ROC curve was higher than 0.7. In addition, it had a good calibration because the *P* value in the calibration test was 0.472 (*P* > .05). These findings demonstrate the accuracy of the model. The changes in sleep phases may indicate that common sleep disturbances after ischemic cerebral infarction, such as prolonged W phase and shortened N3 phase in this study, may be related to PCCI. Our study suggested that cerebral infarcts alter the structure of sleep. Since there are multiple circuits in the territory supplied by the posterior circulation that control sleep phases, this finding is expected, and it is highly speculative that an intervention on sleep phases may have some effect on the incidence of posterior circulation infarctions, which may provide some suggestions for clinical decision-making regarding sleep disturbance after acute ischemic stroke, especially PCCI, including intervention in the W or N3 phase. It may also provide some advice to patients with W or N3 phase disturbance to avoid PCCI effectively, e.g., shortening the W phase or prolonging the N3 phase. To prove this, a prospective study should be conducted as the next step.

This study had some limitations. First, the short study coverage time resulted in few patients being enrolled, and the sleep lab analysis does not exactly reflect the real reality of the “real world”; thus, the results might be skewed. Second, a healthy control group was not comparatively assessed, and subgroup analysis should be performed based on exact infarct location and different sleep patterns to further examine the influence on quality of life, mortality, etc. This study demonstrated that CI causes different degrees of sleep phase disturbances, and the percentages of W and N3 phase disturbances were independent factors associated with PCCI. The former was a risk factor, whereas the latter was a protective factor. This study demonstrated the correlation between cerebral infarction and sleep phase disturbances from a new perspective and provided some suggestions for treatment or prevention decision-making regarding sleep disturbance after CI.

## Acknowledgements

We are grateful to the Stroke Center and Department of Otolaryngology, Baoding First Central Hospital.

## Author contributions

**Conceptualization:** Lu Liu.

**Data curation:** Lu Liu, Nan Gao, Tongle Jia, Liying Geng, Yaning Ma.

**Methodology:** Li Guo.

**Writing – original draft:** Lu Liu.

**Writing – review & editing:** Lu Liu, Weiping Wang.

## Supplementary Material
