# Supplementary material for: Risk factors of disturbed sleep phases to posterior circulation cerebral infarctions: A single-center retrospective study
Source: Medicine (Baltimore). 2023 Oct 13;102(41):e35479. doi: 10.1097/MD.0000000000035479 (PMC10578694; doi:10.1097/MD.0000000000035479)
Supplement: Supplementary file 1 [file medi-102-e35479-s001.docx]

**Table S1.** General information in ACCI and PCCI patients

| Clinical features | ACCI, n=101 | PCCI, n=91 | P |
| --- | --- | --- | --- |
| Sex, n (%) |  |  |  |
| Male | 51(50.5) | 47(51.6%) | 0.886 |
| Female | 50(49.5) | 44(48.4%) |  |
| Age (Yrs), Mean ± SD | 56.25 ± 7.40 | 57.08 ± 6.21 | 0.675 |
| BMI (Kg/m^2^), Mean ± SD | 24.28 ± 3.25 | 26.19 ± 5.45 | 0.38 |
| Past medical history, n (%) |  |  |  |
| Hypertension | 29 (30.2) | 26(27.1) | 0.204 |
| Diabetes | 28(29.2) | 31(32.3) | 0.182 |
| Infarction Location |  |  |  |
| basal ganglia infarction | 67 (69.8) | - |  |
| frontal lobe infarction | 2 (2.1) | - |  |
| internal capsule infarction | 22 (22.9) | - |  |
| temporal lobe infarction | 1 (1.0) | - |  |
| brainstem infarction | - | 60 (62.5) |  |
| occipital infarction | - | 13 (13.5) |  |
| cerebellar infarction | - | 19 (19.8) |  |
| thalamic infarction | - | 4 (4.2) |  |
| multiple location | 4 (4.2) | 3 (3.1) |  |
| Infarction size (cm^2^), Mean ± SD | 1.41 ± 0.78 | 1.91 ± 1.20 | 0.154 |
| Arousal index, Mean ± SD | 35.39 ± 14.97 | 26.33 ± 10.61 | 0.19 |
| Apnoea-hypopnoea index, Mean ± SD | 21.21 ± 17.49 | 24.52 ± 14.85 | 0.689 |
| Periodic limb movement disorder, Mean ± SD | 22.49 ± 15.49 | 18.69 ± 23.81 | 0.694 |
| Epworth Sleepiness Scale, Mean ± SD | 4.39 ± 1.46 | 4.33 ± 1.71 | 0.917 |

ACCI: anterior circulation cerebral infarction; PCCI: posterior circulation cerebral infarction. BMI: Body Mass Index.
